# Coherent coupling between a quantum dot and a donor in silicon

**DOI:** 10.1038/s41467-017-01113-2

**Published:** 2017-10-18

**Authors:** Patrick Harvey-Collard, N. Tobias Jacobson, Martin Rudolph, Jason Dominguez, Gregory A. Ten Eyck, Joel R. Wendt, Tammy Pluym, John King Gamble, Michael P. Lilly, Michel Pioro-Ladrière, Malcolm S. Carroll

**Affiliations:** 1 Département de Physique et Institut Quantique, Université de Sherbrooke, Sherbrooke, QC Canada J1K 2R1; 2 Sandia National Laboratories, Albuquerque, NM 87185 USA; 3 Center for Computing Research, Sandia National Laboratories, Albuquerque, NM 87185 USA; 4 Center for Integrated Nanotechnologies, Sandia National Laboratories, Albuquerque, NM 87185 USA; 5 Quantum Information Science Program, Canadian Institute for Advanced Research, Toronto, ON Canada M5G 1Z8

## Abstract

Individual donors in silicon chips are used as quantum bits with extremely low error rates. However, physical realizations have been limited to one donor because their atomic size causes fabrication challenges. Quantum dot qubits, in contrast, are highly adjustable using electrical gate voltages. This adjustability could be leveraged to deterministically couple donors to quantum dots in arrays of qubits. In this work, we demonstrate the coherent interaction of a ^31^P donor electron with the electron of a metal-oxide-semiconductor quantum dot. We form a logical qubit encoded in the spin singlet and triplet states of the two-electron system. We show that the donor nuclear spin drives coherent rotations between the electronic qubit states through the contact hyperfine interaction. This provides every key element for compact two-electron spin qubits requiring only a single dot and no additional magnetic field gradients, as well as a means to interact with the nuclear spin qubit.

## Abstract (web summary)

In silicon, quantum information can be stored in donors or quantum dots, each with its advantages and limitations—particularly in terms of fabrication. Here the authors coherently couple a phosphorous donor’s electron spin to a quantum dot, encoding information in the hybrid two-electron system’s state.

## Introduction

The silicon industry’s fabrication capability promises to be a differentiating accelerator for the future development of quantum computers built with silicon quantum bits (qubits). Silicon is, furthermore, an appealing material for qubits because it provides an ultra low decoherence environment^1^. In particular, extremely high fidelities have been demonstrated for both the electron^1–5^ and nuclear spins^6^ of a single dopant atom in isotopically-enriched silicon nanostructures^4^. Assembling these exceptional solid-state qubits into a full quantum processor, as first envisioned by Kane^7^, will require coupling donor atoms to one another in a controllable way. This has proven extremely challenging, demanding near-atomic precision in the placement of the donors^8–12^. In contrast, single electron spins confined in quantum dots (QDs)^13–16^ are routinely coupled to one another since quantum dots are highly tunable and fabricated in engineered locations, allowing for controllable and scalable two-qubit interactions^13, 17–20^. For this reason, QDs have been theoretically discussed as intermediates to couple donor qubits^7, 21–25^. Recently, spin blockade has been observed in a silicon QD-donor device^26^. However, the coherent spin coupling between donor- and quantum dot-based qubits has remained elusive. It is the cornerstone advance necessary for exploiting the advantages of these two complementary qubit systems.

Here, we advance silicon-based quantum information processing by coherently coupling a phosphorus donor’s electron spin to a metal-oxide-semiconductor (MOS) QD. In our system, the QD is tuned to few-electron occupancy while simultaneously keeping a nearby donor (D) tunnel-coupled to the QD. The combination of the QD and donor electron qubits gives rise to a joint singlet-triplet (ST) logical encoding analogous to those in double-QD qubits^27, 28^. Specifically, the two logical states are the singlet $\left| S \right\rangle = \left| { \uparrow \downarrow } \right\rangle \! - \! \left| { \downarrow \uparrow } \right\rangle$ and unpolarized triplet $\left| {{T_0}} \right\rangle = \left| { \uparrow \downarrow } \right\rangle \! + \! \left| { \downarrow \uparrow } \right\rangle$. The encoding takes advantage of the contact hyperfine interaction between the donor electron spin and donor nuclear spin. This interaction makes the electron spin on the donor precess at a rate *A*/2 different from the QD electrons, where *A* is the hyperfine coupling strength. The hyperfine interaction thus amounts to an effective magnetic field gradient produced by the single phosphorus nucleus and drives rotations between singlet and triplet states^29^. By electrically controlling the donor charge configuration between ionized and neutral, the rotations can be turned off and on. The electron-electron exchange coupling and the hyperfine interaction with the donor nucleus define two orthogonal control axes for the qubit, and their relative strength is controlled using fast electrical pulses.

The electron qubit formed by the QD-D coupled system is analogous to other ST qubits, while introducing important advantages. It features full electrical control with a uniquely compact design requiring only one QD. The QD-D ST qubit avoids the integration complexities of other Si spin control schemes such as micromagnets^30, 31^, microwave striplines^32, 33^ or additional QDs for full electrical control^14, 34^. The hyperfine coupling to the single nuclear spin introduces a nature-defined and potentially very stable (i.e. low noise) rotation axis for the ST qubit. Furthermore, the system has a natural access to the nuclear spin, which is one of the highest performing solid state qubits^4^. Integration of a coil for nuclear magnetic resonance could enable full control over the nuclear spin qubit. Nuclear spin readout schemes based on ST interactions with the donor have already been proposed^21^, making complete control of these two coupled qubits foreseeable in the near future. The engineered coupling of the QD and D spins constitutes a possible path to realize over nineteen years of different theoretical proposals of donor qubit architectures^7, 22, 24, 25, 35, 36^. For example, the large lithographic quantum dot can facilitate the coupling of neighboring QD-D cells using capacitive coupling^19, 20, 37^ or exchange interaction^13^.

## Results

### Device description

**Fig. 1:**
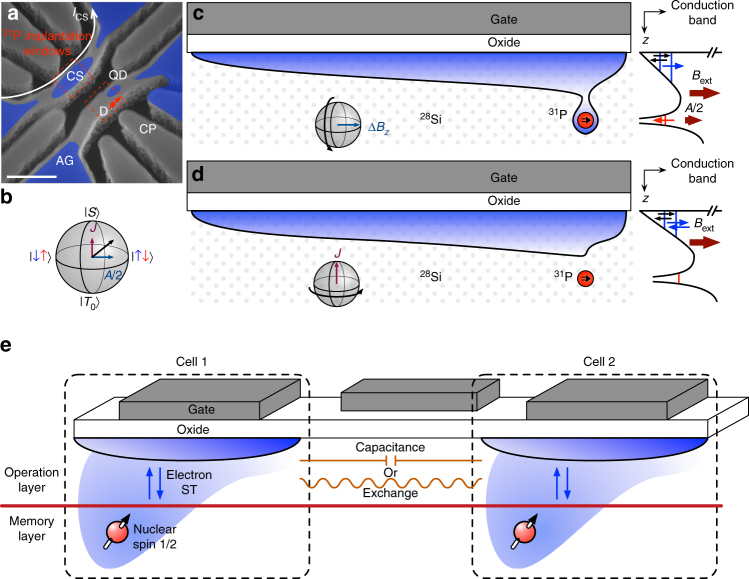
Quantum dot-donor system. **a** Angled-view scanning electron microscope image of the device gate structure. The blue overlay represents the 2D electron gas at the Si-oxide interface. Donors are implanted in the regions designated by the dashed red lines. The relevant donor (D) located next to the quantum dot (QD) is indicated by the red dot. Scale bar: 200 nm. **b** A four-electron filled-shell configuration is used to mimic a two-electron singlet-triplet qubit (see main text for details). The Bloch sphere shows the logical singlet $\left| S \right\rangle = \left| { \uparrow \downarrow } \right\rangle - \left| { \downarrow \uparrow } \right\rangle$ and triplet $\left| {{T_0}} \right\rangle = \left| { \uparrow \downarrow } \right\rangle + \left| { \downarrow \uparrow } \right\rangle$ qubit states at the poles. Electrical voltages adjust the relative magnitudes of *J* and *A*/2: *J* is dominant in the (4, 0) charge configuration, while *A*/2 is in the (3, 1) configuration. **c** S chematic showing the electrons confined in the (3, 1) configuration at the Si-oxide interface in a large tunable QD and separated by a valley splitting, together with the relatively small D potential. The hyperfine interaction with the ^31^P nucleus makes the electrons precess at different rates, creating an effective magnetic field difference Δ*B*_*z*_ = ±*A*/2 between the QD and the D. **d** The donor electron can be moved to the QD using gate voltages. In this (4, 0) configuration, the exchange interaction dominates. **e** Conceptual view of how a coupled QD and D cell could interact with other elements in a future chip. The electron qubit (blue arrows) is well suited for fast operations and readout. The nuclear spin qubit has high coherence and fidelity. Thanks to the large engineered QD, the electron qubits could be coupled through capacitive or exchange interaction without requiring atomic precision in the placement of donors

The QD-D device is fabricated with isotopically-enriched ^28^Si and a foundry-compatible process (i.e. no lift-off processing). We use a poly-silicon gate stack, shown in Fig. 1a, that allows self-aligned ion implantation and subsequent activation annealing process. Phosphorus donors are implanted using the AG gate as a mask. This processing maximizes the probability of placing a D in a suitable location next to the QD. It also facilitates future multi-qubit fabrication that could take advantage of single ion implantation^8^ and a planar QD geometry^24, 25^. Fabrication details are found in the Supplementary Note 1 and are similar to ref. ^38^. A channel of electrons is formed at the MOS interface underneath the wire-shaped accumulation gate (AG) by applying a positive voltage, depicted as a blue overlay in Fig. 1a. Next, a QD island is isolated by applying suitable negative voltages on neighboring gates. A single-electron transistor (SET) is formed in the upper wire to monitor the electron occupation *N* of the QD and the relevant donor, denoted (*N*_QD_, *N*_D_). The SET charge sensor (CS) is also used for spin readout via spin-to-charge conversion. An in-plane magnetic field of 300 mT is applied throughout the experiments and the electron temperature is measured to be 215 mK. Detailed information about fabrication, gate biasing and electron counting is provided in the Supplementary Note 2.

To investigate coherent coupling dynamics between the donor and the QD, we first identify an effective (2, 0) ↔ (1, 1) QD-D charge transition with a total of four electrons, as shown in Fig. 1b–d ^39, 40^. We use the spin filling structure, measured through magnetic fields, to engineer a sufficiently large energy difference *J*_(4,0)_ between the singlet and triplet states^41^, which we observe to be substantially larger for four electrons (~150 μeV) than for two electrons (~60 μeV). Details are available in the Supplementary Note 3. In Si MOS, the valley splitting can be tuned to large values by increasing the electric field perpendicular to the interface, which was verified in this device^42^. Simultaneously keeping the donor in resonance with the few electron QD states, however, constrained the available range of voltage in this design leading to the relatively small two-electron valley splitting. We note two general benefits of using the four-electron configuration: (i) filled shells might be a general approach to circumvent the obstacle of low valley splitting in any material with conduction band degeneracy^14, 43^; and (ii) increased electron numbers can extend the size of the QD due to the increased filling of the potential well, which in turn allows more range in selecting a suitable tunnel coupling to remote donor sites.

### Hyperfine-driven spin rotations

**Fig. 2:**
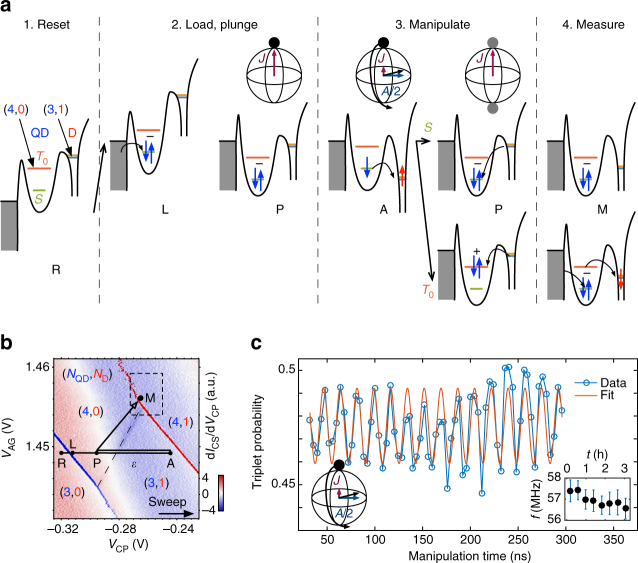
Hyperfine ST rotations. **a** Pulse sequence for the spin manipulations with schematic conduction band diagrams through the reservoir, QD and donor. The system is initialized as (4, 0)*S* and plunged to point P. Then, the detuning is pulsed rapidly to point A, which yields a separated (3, 1)*S*. After a given manipulation time in (3, 1), which rotates the spin between *S* and *T*_0_, it is pulsed back to point P. The state is then either (4, 0)*S* or (4, 0)*T*_0_, and is measured by going to point M, where an enhancement in CS signal occurs (see Supplementary Note 4). **b** QD-D charge stability diagram. Overlaid are the different points of the experiment’s pulse sequence. The detuning $\epsilon$ is defined along the black line with zero detuning at the center of the (4, 0) ↔ (3, 1) transition. **c** Triplet probability vs. manipulation time for $\epsilon$ = 950 μeV. The oscillation frequency is *f* = 56.9 MHz. This is not the bare hyperfine frequency due to a residual exchange of *J*/*h* = 27 MHz, see Fig. 3c. Inset: Frequency of the oscillations for repeated measurements over 3 h. Each point represents data averaged over 22 min, and the error bar represents the 95% confidence interval

Rotations between $\left| S \right\rangle$ and $\left| {{T_0}} \right\rangle$ can be driven by an effective magnetic field gradient Δ*B*_*z*_ = ±*A*/2 between the QD and the donor (in the remainder of the text we will drop the ket notation). These rotations provide a signature of the single ^31^P donor. The source of the effective Δ*B*_*z*_ is the contact hyperfine interaction $A\widehat {\bf{S}} \cdot \widehat {\bf{I}}$ between the donor electron spin $\widehat {\bf{S}}$ and the nuclear spin $\widehat {\bf{I}}$. We expect the nuclear spin state to be projected onto a ±1/2 eigenstate by the repetitive experimental measurement. Rapidly separating a singlet state by pulling one electron onto the donor triggers coherent rotations between the *S* and *T*_0_ states. Reuniting the electrons onto the QD projects the state onto *S* or *T*_0_. We note that spin preparation, manipulations and readout act self-consistently with respect to a fixed but unknown state of the nuclear spin (i.e. the sign of Δ*B*_*z*_) in sufficiently large magnetic fields such that the interaction with the polarized triplets is suppressed (which is the case in this experiment). Moreover, nuclear states are known to be long lived (~seconds) compared to the timescale of electron manipulations^4^, therefore, errors caused by random flips while an electron is on the donor are expected to be negligible. The nuclear state could still have implications for single or multi-qubit operation. In the future, this could be addressed by deterministically setting the nuclear state through various pulsing schemes, such as a single-spin version of dynamic nuclear polarization^44^. To demonstrate the hyperfine-driven rotations, we use the pulse sequence shown in Fig. 2. We prepare a (4, 0)*S* state by first emptying the QD and loading an electron between the singlet and triplet loading lines. Then, we plunge the system at point P (see Fig. 2b). Next, we rapidly separate the electrons by pulsing the system to point A with a 16 ns ramp time. After waiting for a given manipulation time, the system is pulsed back to point P in (4, 0). The ramp time is such that the charge transition is adiabatic, but fast enough to prepare a (3, 1)*S*. Finally, we use an enhanced latching readout developed for this experiment and described in the Supplementary Note 4 to measure the triplet return probability.

Figure 2c shows the triplet return probability as a function of the manipulation time. All the details on the pulse sequence can be found in the Supplementary Notes 5 and 6. We find a ST rotation frequency *f* = 57 MHz. This frequency is the vector sum of the exchange energy *J*($\epsilon$) and *A*/2, such that $hf = \sqrt {{J^2} + {{\left( {A{\rm{/}}2} \right)}^2}}$, where *h* is the Planck constant^29^. We estimate a residual exchange of *J*/*h* = 27 MHz for this detuning from numeric fits to the frequency dependence on detuning (described below). The inset shows that this frequency is very stable over time. Such behavior differs from GaAs systems, for which dynamic nuclear polarization must be used to generate and maintain a particular Δ*B*_*z*_ of similar magnitude^45^. The magnitude and stability of *f* provides a strong indication that the rotations are driven by a single ^31^P. A small and relatively constant frequency drift of around 0.8 MHz is observed over a period of 3.5 h which is consistent with the drift in the electrostatics of the device through the experimentally measured d*J*/d$\epsilon$ relation. Additionally, the observed linewidth is less than natural silicon, which has linewidths greater than 8 MHz for single donors^32^ and is qualitatively consistent with an enriched ^28^Si background. Noise in *J* is believed to presently limit the linewidth, discussed below in terms of $T_2^*$.

### Characterization of exchange interaction

**Fig. 3:**
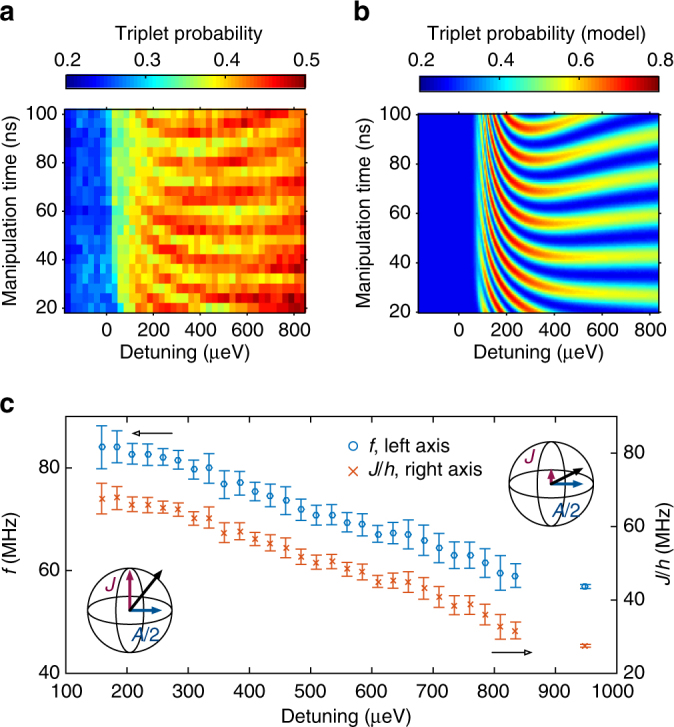
Detuning dependence of exchange. **a**, **b** Experimental **a** and model **b** triplet probability vs. detuning and manipulation time. The oscillations on the time axis are the hyperfine-driven rotations. The phase and shape of the oscillations are well reproduced; however, the mechanisms limiting the visibility are detailed in the Supplementary Note 8. **c** Left axis: Frequency *f* of the oscillations vs. detuning, extracted from fits to **a**. This corresponds to the *S*–*T*_0_ energy gap, allowing a fit for model parameters *t*_S_ = 19 μeV, *t*_T_ = 31 μeV and *A*/2*h* = 50 MHz. Error bars represent the 95% confidence interval. Right axis: Exchange *J* calculated from the frequency after removing the hyperfine contribution $J{\rm{/}}h = \sqrt {{f^2} - {{\left( {A{\rm{/}}2h} \right)}^2}}$

The detuning dependence of the ST rotations reveals additional information about this QD-D system. In Fig. 3a, we plot the triplet return probability against both detuning and manipulation time. As the detuning gets closer to zero, the frequency of the exchange rotations increases, as shown in Fig. 3c. This is consistent with a ST model where the exchange energy *J* between the *S* and *T*_0_ states is not negligible and drives rotations around a tilted axis in the qubit Bloch sphere. To better understand the exact shape of the oscillations of Fig. 3a, we simulate the quantum dynamics of the system using a master equation approach and time-dependent controls. We describe the system using the basis states {(4, 0)*S*, (4, 0)*T*_0_, (3, 1)*S*, (3, 1)*T*_0_}, similarly to previous treatments such as Taylor et al.^29^. The details of the model are given in the Supplementary Note 7. The numerical simulation results are shown in Fig. 3b. The phase and shape of the oscillations is very well reproduced; however, the mechanisms limiting the visibility are numerous and detailed in the Supplementary Note 8. At the moment, we think that addressing the various causes could ultimately produce results on-a-par or even better than state-of-the-art ST qubits. The key fitting parameters of the model are the triplet tunnel coupling *t*_T_, singlet tunnel coupling *t*_S_, and hyperfine interaction *A*. We can determine these parameters using a fit to the data of Fig. 3c, knowing that *hf* equals the energy gap between the *S* and *T*_0_ states. We find *t*_S_ = 19 μeV, *t*_T_ = 31 μeV and *A*/2*h* = 50 MHz. Shifts in *A* of this magnitude relative to the bulk value (of 58.5 MHz, ref. ^32^) have been reported in single donor electron spin resonance (ESR) experiments^4^ and have been attributed to Stark shifts of the contact hyperfine interaction due to the large electric fields in the vicinity of the neighboring QD. The measured value in this work is both consistent with a shallow phosphorus donor and is inconsistent with likely alternatives, such as arsenic. Following the fit procedure, we can extract *J*($\epsilon$) by subtracting the *A*/2 contribution. The result is shown in Fig. 3c.

## Discussion

**Fig. 4:**
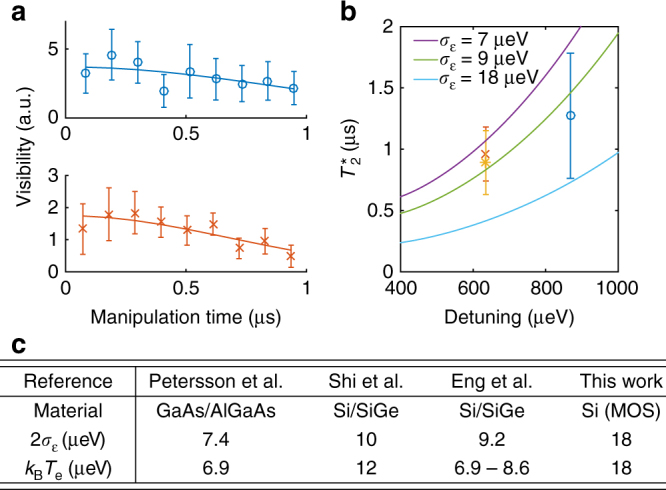
Coherence time. **a** Visibility of ST rotations vs. time and $T_2^*$ fit with a Gaussian decay model for $\epsilon$ = 868 μeV (top) and 635 μeV (bottom). The results are plotted in **b**. Error bars represent the 95% confidence interval. **b** The $T_2^*$ at both detunings is consistent with residual exchange and a detuning noise of ${\sigma _\epsilon }$ = 9 μeV. Error bars represent the 95% confidence interval. **c** Table comparing charge noise in a variety of material systems. All show a similar noise level that seems related to the electronic temperature *T*_e_. *k*_B_ is the Boltzmann constant. References: Petersson et al.^47^, Shi et al.^48^, Eng et al.^14^

Decoherence of MOS QDs^33^ and single donors^4^ has been characterized in separate systems, but the charge noise and magnetic noise properties of strongly hybridized QD-D systems are not well established. Our system provides a unique platform to study these important properties in an effective two-electron case where entanglement is delocalized in the form of a spatially separated singlet or triplet. We measure long time traces and plot the visibility of the oscillations vs. manipulation time *t* in Fig. 4a. The data and method are presented in the Supplementary Note 9. We then fit the decay using a slow detuning noise model that produces a Gaussian decay of the visibility $v = {v_0}\,{\rm{exp}}\left[ { - {{\left( {t{\rm{/}}T_2^*} \right)}^2}} \right]$, where *v*_0_ is an arbitrary initial visibility. We find that $T_2^*$ depends on the detuning (Fig. 4b). To understand this dependence, we use a charge noise model represented by $\epsilon$ noise with a characteristic standard deviation ${\sigma _\epsilon }$ and producing decoherence through *J*($\epsilon$)^46^. Details about the model are given in the Supplementary Note 10. We find that ${\sigma _\epsilon }$ = 9 μeV is consistent with the observed $T_2^*$. In this model, we neglect magnetic noise that could be caused by residual ^29^Si or other sources. Our observations are consistent with $T_2^*$ being limited by charge noise, a mechanism that is expected to play an important role when *J* varies as a function of $\epsilon$ ^46^. We note that 2${\sigma _\epsilon }$ is approximately the electronic temperature *k*_B_ *T*_e_. The noise magnitude has previously been correlated with the electronic temperature^46^. We further tabulate noise magnitudes in a variety of material systems, like GaAs/AlGaAs heterostructures^47^, Si/SiGe heterostructures^14, 48^ and MOS (this work), and show the results in Fig. 4c.

In summary, we have demonstrated coherent coupling between the electrons of two very different qubit systems: a donor atom (natural atom) and a MOS quantum dot (artificial atom^49^). The coherent rotations between the singlet and triplet are driven by a nuclear spin qubit through the contact hyperfine interaction, and produce 10 ns *X*(*π*) rotations with a $T_2^*$ of 1.3 ± 0.7 μs, thus allowing over 100 rotations within the coherence time. A charge noise magnitude of 9 μeV fits the stationary noise model and is a characterization of the MOS interface noise properties, which are found to be of similar magnitude to other common QD material systems. Assuming this model, the $T_2^*$ could possibly be improved by a factor 10 or more by operating at larger detunings where the exchange is negligible, hence taking full advantage of isotopically pure silicon. Our experiments demonstrate the feasibility of using the QD-D system as a compact ST qubit with no additional micromagnets^30, 31^ or QDs (as in all-exchange qubits^14, 50, 51^), and avoid the decoherence mechanisms associated with GaAs or Si host nuclear species^28, 52^. More sophisticated ST qubit control approaches^53, 54^ and optimized preparation/readout parameters will likely increase the visibility and reduce errors of future two-axis QD-D qubit demonstrations. To further speed up the operations compared to the coherence time, it could be possible to use other donor species that have stronger contact hyperfine strengths. Beyond individual ST qubits, this work opens-up compelling possibilities. One such example is the coupling of donor-based qubits without atomic precision placement through, for example, electrostatic coupling between ST qubits^19, 20, 37^. Another example is all-electrical nuclear spin readout^21^ and electric/nuclear magnetic resonance control without high magnetic fields or ESR, thus introducing a nuclear spin qubit as an additional resource.

### Data availability

The authors declare that the data supporting the findings of this study are available within the paper and its Supplementary Information files. Additional data (e.g. source data for figures) are available from the corresponding author upon reasonable request.

## Electronic supplementary material

Supplementary Information
