# Supplementary material for: Coherent coupling between a quantum dot and a donor in silicon
Source: Nat Commun. 2017 Oct 18;8:1029. doi: 10.1038/s41467-017-01113-2 (PMC5715091; doi:10.1038/s41467-017-01113-2)
Supplement: Supplementary file 1 — Supplementary Information [file 41467_2017_1113_MOESM1_ESM.pdf]

SUPPLEMENTARY NOTE 1. DEVICE FABRICATION AND MEASUREMENT

The device used for these experiments is fabricated identically to the one of Fig. 1a. Electrons are confined in a 2D electron gas at the interface between an epitaxial enriched ^{28}Si layer with 500 ppm residual ^{29}Si and a 35 nm gate oxide. Highly n-doped poly-silicon gates (200 nm thick) are patterned on top of the gate oxide using low pressure chemical vapor deposition and plasma etching.[1] These are used to accumulate electrons by applying a positive voltage (in an enhancement mode) or deplete electrons (with negative voltages). Phosphorus donors are implanted in a PMMA resist window that overlaps with the AG gate on both sides of both wires, and the poly-Si gate used as a self-aligned implantation mask. The approximate relevant donor location indicated by the red dot in Fig. 1a of the main text is inferred from various donor-gate capacitance ratios. The source and drain reservoir electrons are connected by n+ regions and ohmic contacts to the instruments. The device is biased to form a SET in the upper wire that is used as a charge sensor (CS), while simultaneously forming a few-electron QD under the lower wire. The CS current I_{CS} is measured using an AC lock-in technique at 403 Hz with 0 DC source-drain bias and 100 μV (rms) AC bias. The derivative with respect to gate voltage is taken numerically to show the QD charge occupancy steps in charge stability diagrams.

SUPPLEMENTARY NOTE 2. FEW ELECTRON REGIME

One can form a clean single QD with this device geometry through biasing that pushes the QD towards one lead, shown in Supplementary Figure 1a. A representative set of gate voltages used for this experiment is shown in Supplementary Figure 1c. We use gates AG and CP to discriminate between QD and D states, respectively. The region where donors interact resonantly with the QD is shown in Supplementary Figure 1b. In this regime, the single QD turns into two strongly coupled QDs in series along the wire axis. This is indicated in the charge stability diagram of Supplementary Figure 1b by two sets of nearly parallel lines. This behavior is systematically reproduced in the devices we measured with such a geometry, which indicates that it is a feature produced by the electrostatics of the device. We can assign occupation numbers to the two QDs, counting from zero. We establish that the QD is in the few electron regime (i.e. emptied) by opening the tunnel barriers to the point where the QD charge-sensed lines become lifetime-broadened without detecting other states (data not shown). Donor and/or defect transitions can be seen cutting through the QD lines and are identified by red lines. These objects anticross with the QD lines in a way analogue to double QDs. The main difference is that they can only accommodate a limited number of charge states, like 0 or 1. We additionally performed magnetospectroscopy[2–4] to verify that the first electron fills as a spin-down electron. For this work, we treat the QD closest to the reservoir as being part of the reservoir itself and neglect its impact on the other QD.

SUPPLEMENTARY NOTE 3. EFFECTIVE (2,0)-(1,1) SYSTEM

To investigate singlet-triplet dynamics, we first identify an effective $(2,0) \leftrightarrow (1,1)$ QD-D charge transition with a total of four electrons, as shown in Fig. 2b. Singlet-triplet states with more than two electrons have been studied theoretically[6] and experimentally[7] in double-QD systems. Using magnetospectroscopy,[2–4] we verify that the QD spin filling is indeed consistent with having a four-electron singlet ground state (see Supplementary Figure 1f). A requirement for efficient spin initialization and readout is that the energy difference $J_{(2,0)}$ between the singlet $(2,0)S$ and triplet $(2,0)T_0$ be much larger than the electron temperature of the experiment,[8] which is 215 mK in this case. Hence charge transitions have a full width at half maximum of approximately 65 μeV. In silicon, the valley splitting is generally the factor limiting $J_{(2,0)}$.[9] In our device and for the values of V_{AG} used, we have measured the valley splitting to be approximately 60 μeV. Consistent with this observation, the two-electron QD states had similarly small values for $J_{(2,0)}$. The four-electron QD state of Fig. 2b, however, has an appreciably larger ST splitting of $J_{(4,0)} = 143$ μeV (as measured from both magnetospectroscopy and excited state spectroscopy). This might be understood as a shell filling effect with QD orbitals, where the pairing of spins allows to circumvent the small valley splitting, as illustrated in the schematic of Fig. 1c-d.[5]

SUPPLEMENTARY NOTE 4. SPIN PREPARATION AND READOUT

We show that we can initialize and read out ST spin states. To do so, we use the pulse sequence of Supplementary Figure 2a. The system is initialized into a $(4,0)S$ or $(4,0)T$ state (where T stands for any triplet) by first ejecting the fourth electron at point R (as defined in Fig. 2b), and then loading either a singlet (S) or triplet (T) state by carefully tuning the load level of point L. A deeper load tends to prepare T states due to their ~ 10 times faster loading rate. After passing through an intermediate point P, which will be important for spin manipulations, the gate voltages are pulsed to point M for spin readout. The readout mechanism is shown in Supplementary Figure 2b. Through Pauli-blockade, the spin state is converted to either a $(4,0)$ or $(4,1)$ charge state depending on whether the initial spin state was a singlet or a triplet, respectively. The mechanism relies on a charge hysteresis effect caused

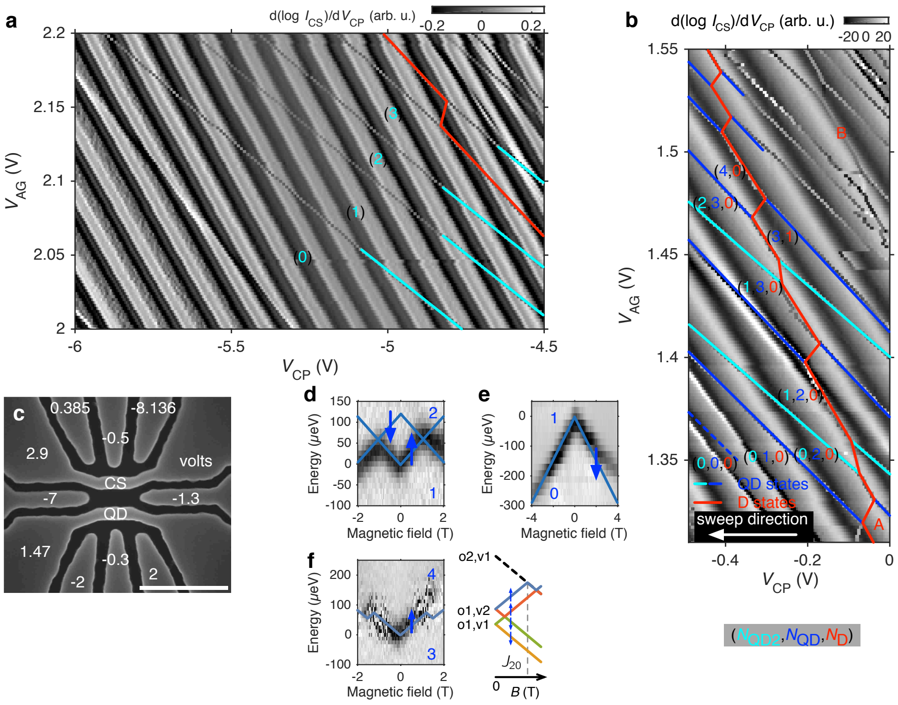

Supplementary Figure 1. Quantum dot and donor states. (**a**) Charge stability diagram in the single dot regime. The broad background features are the successive Coulomb peaks of the SET charge sensor (CS). The sharp features indicated by colored lines are the QD and donor charge transitions. (**b**) Charge stability diagram showing all states. Notation: $(N_{\mathrm{QD2}}, N_{\mathrm{QD}}, N_{\mathrm{D}})$. The QD2 represents a QD that is strongly coupled to the lead and is ignored in the main text. The red lines represent donor or defect states cutting through the QD states. These anti-cross with the QD in a double-QD honeycomb fashion but have only 0 or 1 electron occupancies possible. (**c**) Typical gate voltages for the experiment. (**d-e**) Opening the QD-lead tunnel barriers allows to probe the spin filling of the first two QD states through magnetospectroscopy. CP gate voltage has been converted to energy using a lever arm. (**f**) Magnetospectroscopy data showing the $(3,0) \leftrightarrow (4,0)$ transition loading as a spin singlet. The $(4,0)$ ground state hence forms an effective $(2,0)S$ with exchange splitting $J_{(4,0)} = 143$ μeV (confirmed with pulse spectroscopy). The CP gate voltage was converted to energy E through a lever arm. Grey scale: $\mathrm{d}I_{\mathrm{CS}}/\mathrm{d}E$ (arb. u.). Right schematic: The observed spin filling is qualitatively consistent with a simple shell filling model (see e.g. Ref. 5). The states have a valley-like (v1 and v2) or orbit-like (o1 and o2) character.

by the absence of direct access to a charge reservoir for the donor (Supplementary Figure 2c). Hence, the donor $\leftrightarrow$ lead transitions are very slow because they have to go through a co-tunneling process to equilibrate.[10] Placing point M between the S and T charge preserving transitions then allows a fast relaxation path to the charge ground state only if the initial state was $(4,0)T$. If the state was $(4,0)S$, the system is locked in a metastable charge configuration. The resulting CS signal is enhanced because the final charge configuration differs by one electron and lasts longer than the relaxation time of the $(4,0)T$ state. A charge enhancement effect like this has been previously highlighted by Studenikin *et al.*[11,12] This readout mechanism allows us to use averaged measurements instead of single-shot. Since the measurement step is the longest in the pulse sequence, the current at point M in Supplementary Figure 2d-e is proportional to the triplet probability. All state measurements throughout this work are averaged over many (150 to 200) cycles. Details about the pulse sequence, loading rates, relaxation rates and probability calibration are given in the next section.

SUPPLEMENTARY NOTE 5. PULSE SEQUENCE, LOADING AND RELAXATION RATES

The AC component of pulses in the experiment is applied using an Agilent 33500B arbitrary waveform generator using two synchronized channels for the AG and CP gates. The waveform is composed of DC and AC components and applied to the gates through a room temperature bias tee. The waveforms are applied such that all target points are fixed in the charge stability diagram, except the ones explicitly varied for a particular measurement (e.g. manipulation time or position of point M). The $(4,0)S$ loading rate is approximately $1/(60$ μs$)$, and the $(4,0)T$ loading rate approximately $1/(6$ μs$)$. The $(4,0)T - (4,0)S$ relaxation time is approximately 375 μs, determined by preparing mostly $(4,0)T$ and measuring the triplet probability decay versus time. The metastable state lifetime is roughly 2 to 4 ms. We define zero detuning (the energy difference ϵ between the QD and D) at the QD-D charge transition, and positive detunings along

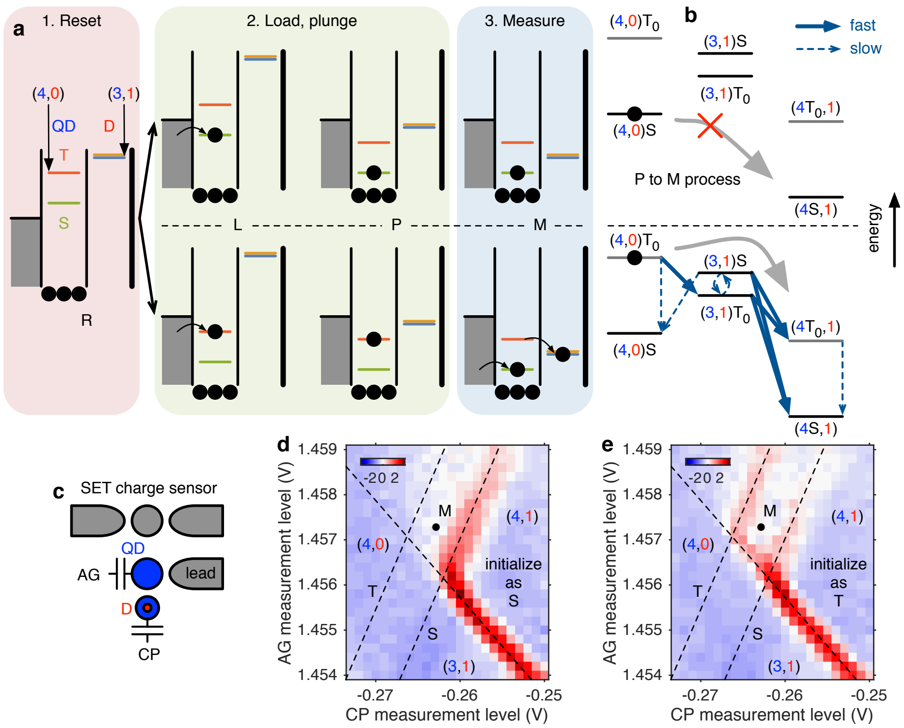

Supplementary Figure 2. Spin preparation and readout. (**a**) Measurement pulse sequence. The state is reset by emptying the QD at point R (see Fig. 2b). A $(4,0)S$ or $(4,0)T$ is loaded at point L. After passing through point P, the system is biased to point M for readout. (**b**) Readout process at point M. If the state was $(4,0)S$, the system is locked in a metastable charge state. If the state was $(4,0)T$, the pulse is beyond the $(4,0) \leftrightarrow (3,1)$ transition line, which unlocks the relaxation to $(4,1)$ by going through $(3,1)$. (**c**) Schematic showing the QD-D configuration. The D has no direct connection to a lead, which causes the hysteresis. (**d-e**) Readout demonstration. The coordinates of point M (only) are swept across the charge stability diagram. When preparing predominantly singlet (S) states, as in (D), only the S QD-D charge transition is visible. In contrast, preparing predominantly triplet (T) states reveals the T QD-D charge transition, as in **e**. The CS current at the location labeled M is then proportional to the triplet probability. Color scale: $\mathrm{d}I_{\mathrm{CS}}/\mathrm{d}V_{\mathrm{CP}}$ (arb. u.).

Supplementary Table 1. Pulse sequence parameters. Table of pulse sequence points (as defined in main text Fig. 2b), ramp time to point (from previous point), and wait time at point, for a typical manipulation pulse sequence used. The sequence is played in a loop.

Point	Ramp time (µs)	Wait time (µs)
R	10	50
L	0.1	150
P	1	0.2
A	0.016	0.1
P	0.016	0.2
M	10	350

V_{CP} in the $(3,1)$ direction with a 17 µeV mV^{-1} lever arm. Then, we plunge the system to $\epsilon = -250$ µeV at point P (see Fig. 2b). Next, we rapidly pulse the system to $\epsilon = 950$ µeV (point A, Fig. 2) or a variable detuning (Fig. 3) with a 16 ns ramp time. After waiting for a given manipulation time, the system is pulsed back to point P in $(4,0)$.

SUPPLEMENTARY NOTE 6. PROBABILITY CALIBRATION

To calibrate the triplet probability, the following procedure is used. First, the CP gate voltage of the measurement point M and the loading point L are swept to tune the readout and initialization, respectively, using the same waveform as for state manipulation except for point A (such that no manipulations are done). The resulting CS current is mapped in Supplementary Figure 3a. Given a certain load level, the CS current is then plotted versus CP measurement level, Supplementary Figure 3b. The current has a downward linear trend because of the CS Coulomb peak flank and a step that is similar in origin to a normal charge sensing signal. To the left of the measurement window the current always corresponds to a singlet signal, and to the right it always corresponds to a triplet signal. By extrapolating what this current would be assuming a linear background, one can determine what the pure singlet and triplet signals should be in the measurement window. The actual triplet probability is determined using a linear transformation that maps I_{CS} to triplet probability. When manipulations are

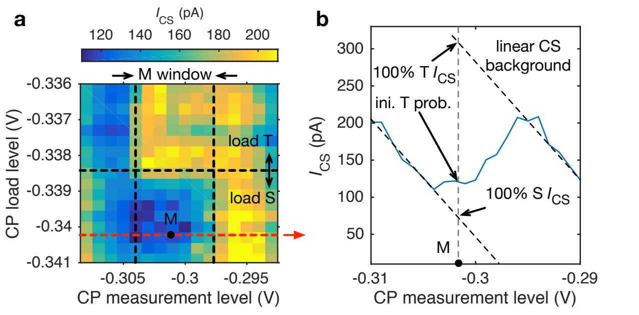

Supplementary Figure 3. Probability calibration. (**a**) The CP gate voltage of measurement point M and loading point L are swept to tune the readout and initialization, respectively, using the same waveform as for state manipulation except for point A (no manipulations). This method allows to map the measurement and initialization windows. (**b**) A cut through measurement levels reveals what the current would be for pure singlet (S) or triplet (T) states in the measurement window.

performed, the duty cycle of the waveform is changed by at most 0.2%, so the calibration is largely unaffected. Any systematic error introduced by this method (e.g. broadening of transitions due to temperature) would tend to underestimate the visibility of oscillations.

SUPPLEMENTARY NOTE 7. SINGLET-TRIPLET DYNAMICS MODEL

We model the ST system with a 4×4 Hamiltonian in the basis $\{|(4,0)S\rangle, |(4,0)T_0\rangle, |(3,1)S\rangle, |(3,1)T_0\rangle\}$ given by

$$H(t) = \frac{1}{2}\begin{pmatrix} \epsilon(t) & 0 & -t_{\mathrm{S}} & 0 \\ 0 & 2J_{(4,0)} + \epsilon(t) & 0 & -t_{\mathrm{T}} \\ -t_{\mathrm{S}} & 0 & -\epsilon(t) & -A/2 \\ 0 & -t_{\mathrm{T}} & -A/2 & 2J_{(3,1)} - \epsilon(t) \end{pmatrix}, \tag{1}$$

where $J_{(4,0)}$ and $J_{(3,1)}$ are the exchange between singlet and triplet states in the $(4,0)$ and $(3,1)$ charge sectors, respectively, t_{S} (t_{T}) is the QD-D tunnel coupling for the singlet (triplet) states, $A/2$ is the effective magnetic field gradient due to the contact hyperfine interaction in the $(3,1)$ configuration, and $\epsilon(t)$ is the detuning. We fix $J_{(4,0)} = 143$ μeV and $J_{(3,1)} = 0$ μeV. For a given control schedule $\epsilon(t)$, we numerically integrate to solve for the time evolution of the density matrix $\rho(t)$ generated by the von Neumann equation

$$\frac{\mathrm{d}\rho(t)}{\mathrm{d}t} = -\frac{i}{\hbar}[H(t), \rho]. \tag{2}$$

To model the effect of finite control bandwidth, the pulse sequence we consider in our numerical simulations is given by the ideal pulse sequence after having been filtered through a (low pass) RC filter,

$$\tilde{\epsilon}(t) = \int_{-\infty}^{\infty} \mathrm{d}\tau h_{\mathrm{RC}}(\tau)\epsilon(t-\tau), \tag{3}$$

where $h_{\mathrm{RC}}(\tau) = \frac{1}{RC}\theta(t)\,\mathrm{e}^{-t/RC}$ is the impulse response function and $\theta(t)$ is the Heaviside step function. Considering various filtered control schedules $\tilde{\epsilon}(t)$, we find that a time constant $RC = 10$ ns is consistent with the experiment.

SUPPLEMENTARY NOTE 8. FACTORS LIMITING THE VISIBILITY

In the main text Fig. 2c, the visibility of the coherent rotations is low. This discussion identifies the different contributions to the visibility. It should be noted that the rotations are approximately 100 times faster than the coherence time. Therefore, the fidelity of the rotation itself should be quite high. Factors contributing to the reduced visibility are state preparation and measurement (SPAM) errors, additional incoherent or leakage processes during the fast ramp in/out of the $(3,1)$ region, and the control protocol itself. It should be noted that the control protocol used is not expected to produce full visibility according to our simulations. This is in part due to the limited bandwidth of the pulse in this setup (i.e. part of the wavefunction remains in the ground state because of partial adiabatic transfer of the spin state in the strong gradient field). Through various measurements we estimate that preparation errors alone are responsible for the 24% triplet probability background in the $(4,0)$ region and limit the visibility to $(1 - 0.24 \times 2) = 52\%$. Singlet preparation was limited by the slow QD-lead tunnel rate which required long loading steps that were competing with the bias tee time constant. The readout process could also yield additional errors at the $\sim 15\%$ and $\sim 30\%$ levels for singlets and triplets respectively due to triplet relaxation and various technical compromises. The dynamics model in the main text takes preparation errors into account and predicts a visibility of approximately 30%, which is the simulated data shown in the main text. Adding measurement errors further reduces the expected visibility to $\sim 17\%$. In the main text data, the visibility is around 6%. This additional loss of visibility is dominated by an error process that occurs when the zero-detuning line is crossed. The exact mechanism is unknown. We speculate that it could be due to incoherent charge excitation/relaxation near the zero detuning point itself. If this is the case, a faster pulsing rise time and a bigger tunnel coupling would be expected to reduce errors.

SUPPLEMENTARY NOTE 9. COHERENCE TIME ANALYSIS

To extract the visibility v of the ST oscillations of Fig. 4a, the following method is employed. The source

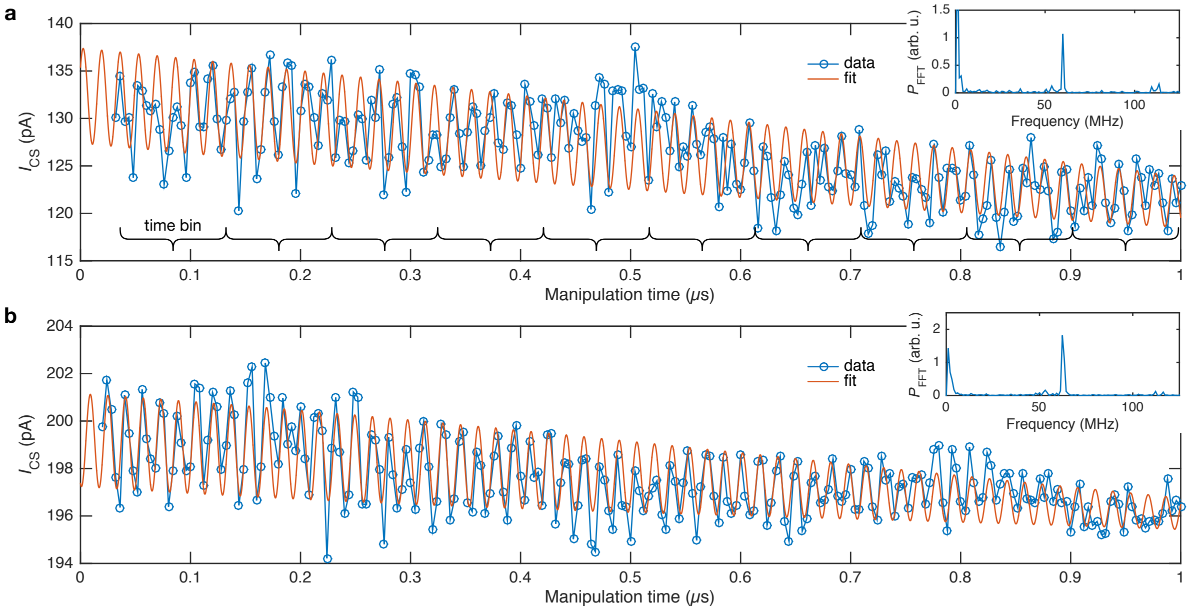

Supplementary Figure 4. Long singlet-triplet rotations. (**a-b**) Singlet-triplet oscillations used to extract the visibility and T_2^*. The charge sensor current $I_{\rm CS}$ is proportional to the triplet probability. The visibility as a function of time is determined by the amplitude of a sine fit for each time bin separately (not shown). The overlay fit curve is a sinusoidal fit with Gaussian decay that has good agreement with the data and the time binning method. In (a), $\epsilon = 868$ μeV and the fit yields $T_2^* = 1.3 \pm 0.7$ μs. The error is the 95% confidence interval. In (b), $\epsilon = 635$ μeV and the fit yields $T_2^* = 0.96 \pm 0.31$ μs. Insets: Fast Fourier Transform power ($P_{\rm FFT}$) spectrum showing a clear single-frequency signal.

data is shown in Supplementary Figure 4. Because of CS drift over the long periods of time required to acquire these longer time traces (2 hours each in this case), the CS current (proportional to triplet probability) has a general downward trend and some residual fluctuations. To remove these fluctuations and smooth the data, the time trace is divided into time bins of approximately 100 ns. The oscillations in each time bin are fitted with a sine function of fixed frequency. The amplitude for each time bin is then reported as visibility in Fig. 4a. The visibility decay is then fitted using a Gaussian decay, as detailed in the "Detuning noise model" section. We have verified that this time binning method agrees well with other methods such as maximum likelihood analysis. We now look at the apparent modulations of the oscillations in Fig. 2c of the main text and Supplementary Figure 4. These are believed to arise from the averaging of a limited ensemble of traces with slightly different frequencies. This is expected because of the slow charge noise and light drift, and leads to beating-like features. We also calculate the Fourier transform of the data to verify the spectral content of the signal and find a single large peak at the expected frequency.

SUPPLEMENTARY NOTE 10. DETUNING NOISE MODEL

Since our device is fabricated with enriched ^{28}Si, the fluctuations in the "magnetic" control axis $A/2$ are expected to be small. Other work in ST qubits has shown that a dominant mechanism limiting the coherence is noise in exchange $J(\epsilon)$ induced by quasi-static noise on the detuning $\epsilon \rightarrow \epsilon + \eta$,[13,14] i.e. "charge" noise. Given a quasi-static noise on the detuning η having zero mean and standard deviation σ_ϵ, an ensemble average leads to a Gaussian decay of the coherence of the form

$$C(t) = \int_{-\infty}^{\infty} \mathrm{d}\eta P(\eta) \cos\left(\frac{t\Delta(\epsilon+\eta)}{\hbar}\right) \tag{4}$$

$$= \exp\left[-\left(\frac{t}{T_2^*}\right)^2\right] \cos\left(\frac{t\Delta(\epsilon)}{\hbar}\right), \tag{5}$$

where $P(\eta) = \mathrm{e}^{-\eta^2/2\sigma_\epsilon^2}/\sqrt{2\pi}\sigma_\epsilon$, $\Delta(\epsilon)$ is the energy gap $\Delta(\epsilon) = \sqrt{J^2 + (A/2)^2}$, and

$$T_2^* = \frac{\sqrt{2}\hbar}{\sigma_\epsilon \left|\partial\Delta/\partial\epsilon\right|}. \tag{6}$$

Since the values we report for T_2^* pertain to an ensemble average of measurements over a timescale of hours, our estimated detuning noise strength includes the effects of a secular drift component as well. While sufficiently large variations of the detuning can lead to Stark shifting of the contact hyperfine strength A, this Stark shifting effect should be small compared to the $\sigma_\epsilon \sim 9$ μeV that we observe.

SUPPLEMENTARY REFERENCES

1. Tracy, L. A. *et al.* Electron spin lifetime of a single antimony donor in silicon. *Applied Physics Letters* **103**, 143115 (2013).
2. Tarucha, S., Austing, D. G., Honda, T., van der Hage, R. J. & Kouwenhoven, L. P. Shell filling and spin effects in a few electron quantum dot. *Phys. Rev. Lett.* **77**, 3613–3616 (1996).
3. Potok, R. *et al.* Spin and polarized current from Coulomb blockaded quantum dots. *Phys. Rev. Lett.* **91**, 016802 (2003).
4. Xiao, M., House, M. G. & Jiang, H. W. Parallel spin filling and energy spectroscopy in few-electron Si metal-on-semiconductor-based quantum dots. *Applied Physics Letters* **97**, 032103 (2010).
5. Lim, W. H., Yang, C. H., Zwanenburg, F. A. & Dzurak, A. S. Spin filling of valley–orbit states in a silicon quantum dot. *Nanotechnology* **22**, 335704 (2011).
6. Nielsen, E., Barnes, E., Kestner, J. P. & Das Sarma, S. Six-electron semiconductor double quantum dot qubits. *Phys. Rev. B* **88**, 195131 (2013).
7. Higginbotham, A. P., Kuemmeth, F., Hanson, M. P., Gossard, A. C. & Marcus, C. M. Coherent operations and screening in multielectron spin qubits. *Phys. Rev. Lett.* **112**, 026801 (2014).
8. Johnson, A. C. *et al.* Triplet-singlet spin relaxation via nuclei in a double quantum dot. *Nature* **435**, 925–928 (2005).
9. Lai, N. S. *et al.* Pauli spin blockade in a highly tunable silicon double quantum dot. *Sci. Rep.* **1**, 110 (2011).
10. Yang, C. H. *et al.* Charge state hysteresis in semiconductor quantum dots. *Applied Physics Letters* **105**, 183505 (2014).
11. Petersson, K. D., Petta, J. R., Lu, H. & Gossard, A. C. Quantum coherence in a one-electron semiconductor charge qubit. *Phys. Rev. Lett.* **105**, 246804 (2010).
12. Studenikin, S. A. *et al.* Enhanced charge detection of spin qubit readout via an intermediate state. *Applied Physics Letters* **101**, 233101 (2012).
13. Dial, O. E. *et al.* Charge noise spectroscopy using coherent exchange oscillations in a singlet-triplet qubit. *Phys. Rev. Lett.* **110**, 146804 (2013).
14. Bertrand, B. *et al.* Quantum manipulation of two-electron spin states in isolated double quantum dots. *Phys. Rev. Lett.* **115**, 096801 (2015).
